# Passive earth pressure analysis considering hydraulic and mechanical hysteresis for unsaturated soil

**DOI:** 10.1371/journal.pone.0310536

**Published:** 2025-01-17

**Authors:** Bestun J. Shwan

**Affiliations:** Water Resources Engineering Department, College of Engineering, Salahaddin University, Erbil, Iraq; Instituto Tecnologico de Aeronautica, BRAZIL

## Abstract

This paper addresses the mechanical characteristics of a passive earth pressure problem taking into account water retention curve (SWRC) hysteresis. Both hydraulic (drying and wetting cycles) and mechanical hysteresis were considered. Parametric studies were carried out at various air entry values (*AEV* = 5–30 kPa), different wall frictions (δ = 0, 0.33 *ϕ*, 0.5 *ϕ*, 0.67 *ϕ* and δ = *ϕ*), and unsaturated conditions (covering the zone from the fully saturated to the transition suction, 0 - ~40 kPa) using an upper bound theorem. The numerical results were compared with a passive earth pressure equation based on the limit equilibrium method. The results indicated significant influences of SWRC hysteresis and wall frictions on the passive thrust (*P*_*p*_) for the modeled suction profiles. An increase of 1.31-fold in *P*_*p*_ was obtained when the *AEV* changed from 10 to 20 kPa at the water table (H_w_) = -2 m. Additionally, the combined effects of *AEV* and δ were found to be significant. An increase of 4.14-fold in *P*_*p*_ at H_w_ = -3 m was obtained when comparing the case of *AEV* = 30 kPa and δ = *ϕ* with *AEV* = 5 kPa and δ = 0. Based on the parametric studies, a series of design charts of the exerted passive thrust were proposed.

## 1. Introduction

Retaining structures have been comprehensively studied and applied in practical fields, e.g., [1–3]. However, these studies mainly considered fully dry and saturated conditions, without taking into account the influence of various saturation conditions. However, backfill materials are inevitably exposed to various climatic conditions, including drying and wetting cycles, which lead to an unsaturated state. Therefore, extensive attempts have been made to investigate the influence of two main stress state variables, suction and degree of saturation, on soil strength in general, [4, 5] and specifically on the passive earth pressure, e.g. [6–13].

Vo and Russell (2014), for example, extended the slip line theory to account for unsaturated conditions using an effective stress equation. The theory was applied to a wall problem accounting for the soil-wall friction. The analysis was carried out under two saturation conditions: steady state evaporation and infiltration. It was found that suction can increase the passive stress, affected by the flow type. Shwan [9, 10] carried out parametric studies to investigate the effect of suction stress on total passive earth pressure for sandy soils. The results showed significant gain in passive thrust, owing to the unsaturated conditions. Additionally, Shahrokhabadi et al. (2019) conducted a numerical study to investigate the effect of transient infiltration on passive earth pressure. The numerical results revealed nonlinear characteristics in passive pressure, attributed to the transient infiltration. While, Deng and Yang (2019) developed an analytical method to consider the effect of suction on the passive earth pressure. The analysis, based on the limit equilibrium method and plastic soil mass, showed that passive earth pressure can be significantly affected by unsaturated conditions. Finally, Fathipour et al. (2020) studied the influence of unsaturated conditions on passive earth pressure using an analysis based on lower bound limit analysis coupled with the finite element method and the second-order cone programming. The numerical study considered several factors such as soil type, effective internal friction angle, wall roughness, and soil anisotropy. It was deduced that suction stress resulted in higher passive earth pressure.

The aforementioned studies considerably aimed to enhance the understanding and assist in the design of retaining structures. However, there is still a need to further extend the comprehension of other well-known unsaturated features, such as hydraulic (drying and wetting cycles) and mechanical (stress-state) behavior. These features have been successfully correlated with the soil water retention curve (SWRC), see [14–17].

The water retention capacity of soils is significantly influenced by various factors, such as loading, unloading, swelling, and shrinkage, (2011), as well as the drying and wetting cycles, (2008). Therefore, the retention behavior changes considerably. As a result, the retention behavior undergoes substantial changes, causing a shift in the SWRC position. During a wetting cycle and under null loading conditions, the wetting path diverges from the drying path due to the hysteresis of the SWRC. Consequently, the SWRC exhibits hysteresis in response to wetting and drying cycles, a well-documented phenomenon in the literature known as hydraulic hysteresis, which significantly influences soil behavior.

In addition, under load application schemes, the retention capacity can also be affected due to the stress-strain behavior or mechanical hysteresis, Romero (1999). The hydraulic and mechanical hysteresis, therefore, cause a shift in the position of the SWRC. This results in a change in the position of the air entry value. The mechanical hysteresis not only causes a shift in the position of the SWRC but also changes its slope, [18, 19].

The effect of hydraulic and mechanical hysteresis has been correlated with shear strength, e.g., [20–22]. Rahardjo et al. (2004) observed a variation in shear strength due to hydraulic history. Khoury and Miller (2012) reported higher shear strength for specimens subjected to a wetting path after drying, compared to those subjected to only a drying path. This finding has been confirmed by others, such as [23, 24]. More recently; Khosravi et al. (2018) [25] reported that, upon drying and wetting, the stiffness of unsaturated sand exhibited oscillations in conjunction with matric suction and degree of saturation. However, lower stiffness was observed during wetting compared to the values recorded during the drying phase.

SWRC hysteresis has been extensively studied in previous research, such as those conducted by [26–32]. Various constitutive models have been proposed, e.g. [33–37]. Despite the comprehensive examination of these features, there remains a notable gap in the literature concerning the application of SWRC hysteresis in the analysis of passive earth pressure.

This paper addresses this gap by investigating the effect of SWRC hysteresis on passive earth pressure. The study considers different suction profiles and soil-wall frictions for a rigid concrete retaining wall. A parametric study is carried out using a modified upper bound theorem, UNSAT-DLO approach, where the numerical results are validated against an extended Rankine equation based on the equilibrium limit method. The equation extended by Fredlund and Rahradjo (1993) was formulated to account for unsaturated conditions. This study aims to propose a set of design charts for retaining structures that account for hydraulic and mechanical hysteresis, essential for engineering design considerations in the various scenarios stated above.

## 2. Water retention curve hysteresis

As stated above, hysteresis can induce a curve shift, either through hydraulic (drying and wetting cycles) or due to stress-state behavior. Sugii (2002) reported a shift of 27 kPa in suction on the drying path for a loam, corresponding to a decrease in void ratio from 0.78 to 0.63 at approximately degree of saturation (*S*_*r*_) = 0.76. Romero (1999) also observed a shift in SWRC position on the drying path for a Boom clay of about 400 kPa at *S*_*r*_ ≈0.96 when void ratio decreased from 0.93 to 0.59. Similar mechanical hysteresis, indicating the evolution of air entry value with void ratio, were reported by [15, 17, 38–41]. The experimental evidence presented above clearly demonstrates the significant impact of hydraulic and mechanical hysteresis on shifting the position of the SWRC, thereby influencing soil behavior.

**Fig 1:**
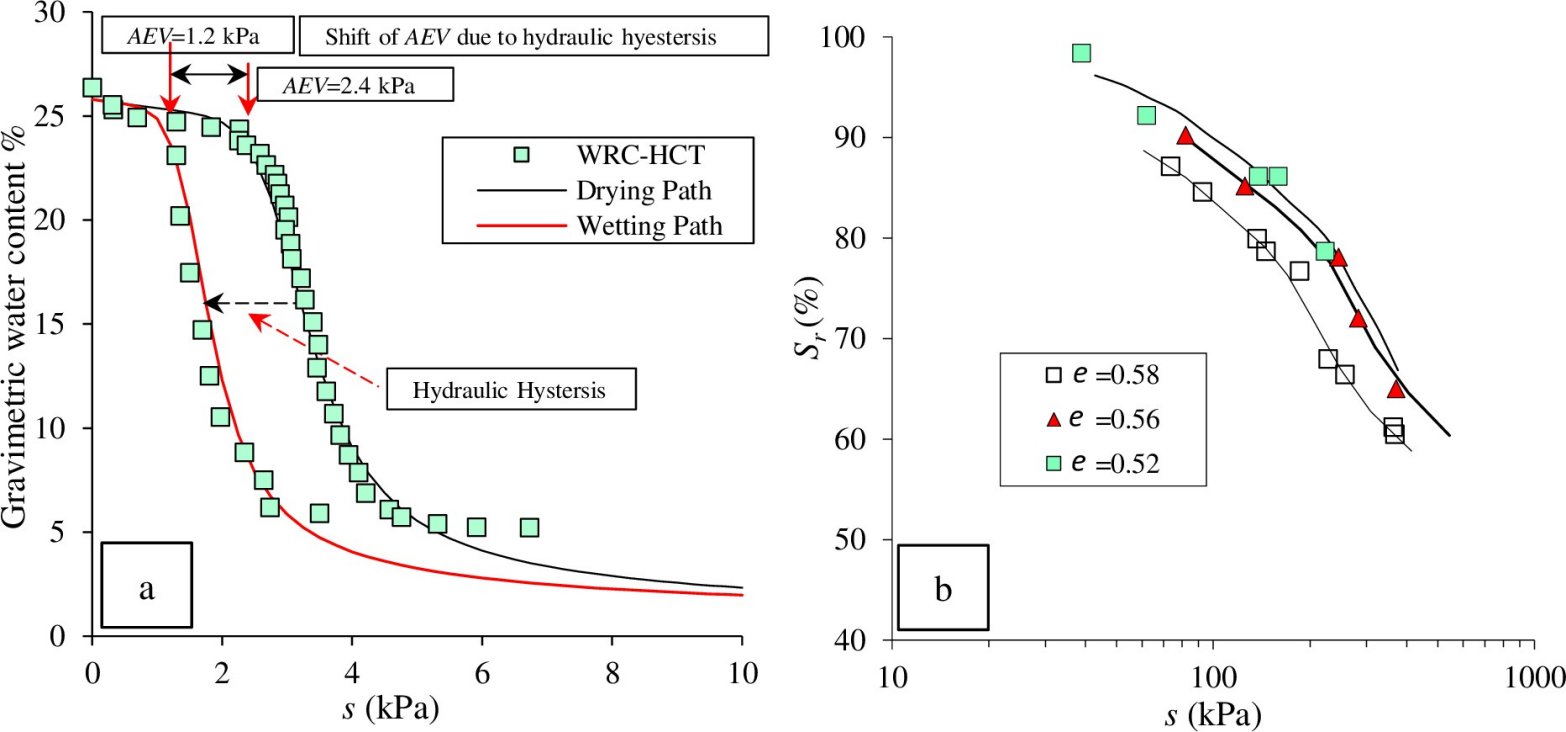
(a) hydraulic hysteresis for an SWRC for a fine sand, after (2015) and (b) mechanical hysteresis for an SWRC for a clay, after (1999).

Fig 1A and 1B present supporting evidence from the literature in line with the aforementioned experimental evidence above. In Fig 1A, an SWRC for a fine sand, obtained by Shwan [42] using the hanging column technique (HCT) with no load application, is depicted. The SWRCs were obtained during the drying phase, after which the sample underwent a wetting cycle. The noticeable change is evident, indicating approximately a 50% reduction in the air entry value (*AEV*) due to hydraulic hysteresis. Fig 1B displays various SWRCs at different void ratios (e) for a clay soil. The influence of the initial void ratio on the mechanical hysteresis of the SWRC is apparent. Specifically, a smaller void ratio corresponds to a higher applied suction within the applied capillary range.

**Fig 2:**
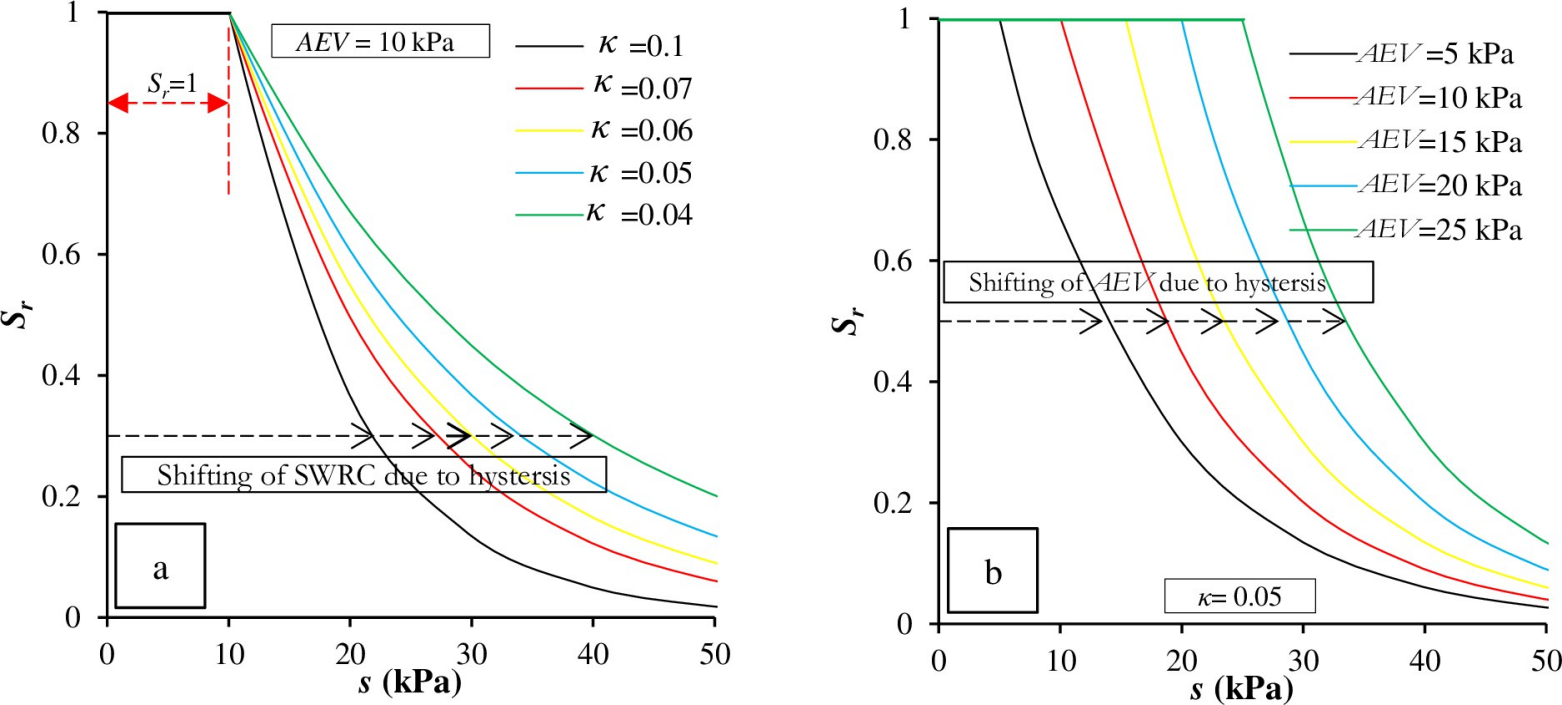
SWRCs for a simulated soil considering (a) hydraulic hysteresis and (b) volumetric hysteresis.

Therefore, the consideration of hydraulic (drying and wetting cycles) and mechanical hysteresis effects on passive earth pressure is included in this study in a simple manner, for a simulated soil subjected to both hydraulic and mechanical hysteresis. Fig 2A and 2B show various SWRCs plotted using Eq 1, as suggested by Shwan and Smith [43].

$$S_{r}=e^{-\kappa (s-AEV)}$$ (1)

where *e* is the exponential function (= 2.718), *κ* is a fitting parameter (kPa^-1^) considers hydraulic and mechanical hysteresis (discussed in the next paragraph, also see [19, 44]), and *s* is the suction (kPa). The use of the exponential function for SWRC was also proposed in the literature, e.g., [45]. Further validation of Eq 1 can found by [46, 47].

Representation of the hydraulic and mechanical hysteresis were plotted using various *κ* at a constant *AEV* and vice versa as shown in Fig 2A and 2B, respectively. The SWRCs comprise five different *κ* and *AEV* to represent the change in SWRC position in two ways: where the position of the *AEV* is constant or changeable. It is clear that Fig 2A and 2B show two different trends, where the main change in retaining water within the particle pores differs. Fig 2A displays various SWRCs where a significant change in *S*_*r*_ occurs in the transition zone, defined between *s* = 10 kPa and = 40 kPa. In Fig 2B; the primary change is represented by shifting the position of *AEV* for the SWRCs, where the slope (shape) of the curves is constant. In both cases, there is an obvious shift of the position of the curve (*κ* = 0.1) or *AEV* = 5 kPa, as indicated by the arrows. The change in the SWRC position is mainly attributed to hydraulic and mechanical hysteresis.

**Fig 3:**
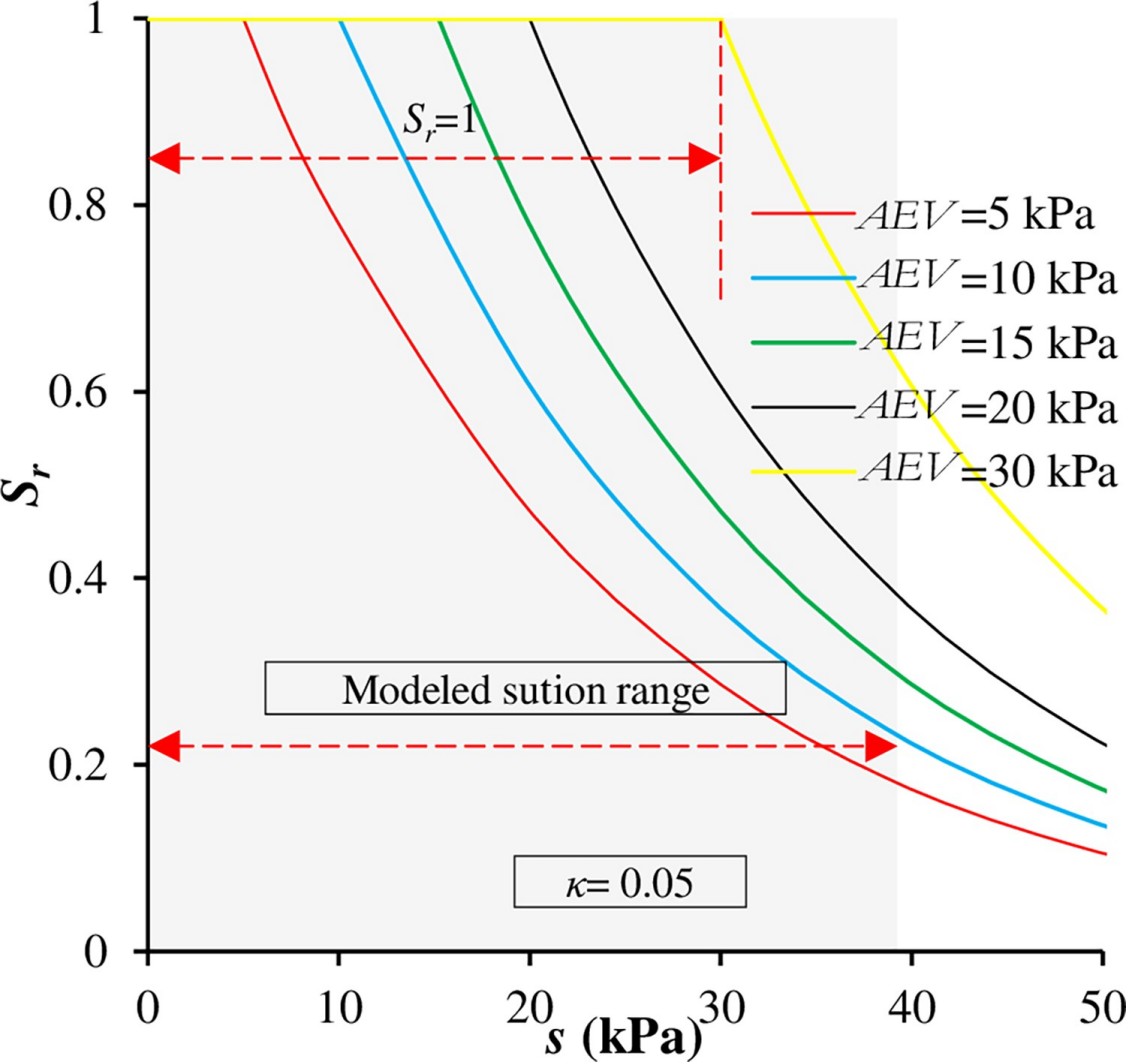
SWRC for the simulated soil considering hydraulic/volumetric hysteresis.

It appears from Fig 1A and 1B that, in both cases, the position of *AEV* is variable. Therefore, this study took into consideration the shift in *AEV* position to represent hydraulic and mechanical hysteresis. The simulated soil is plotted at different *AEV* and constant *κ* = 0.05, as shown in Fig 3. The maximum shift between the suggested *AEV* values was 25 kPa, which is close the experimental results obtained by Sugii (2002) (27 kPa), as stated previously. The range of suction modeled was from 0 to about 40 kPa, ensuring coverage of the transition zone. Full saturation was assumed for any *s* ≤ *AEV* in the analysis.

It should be noted that *AEV* varies considerably with particle size. Shwan [48] determined SWRCs for ten sands using the filter paper method, with the sands classified as coarse to fine particles. The obtained values of *AEV*s ranged from less than 1 to ≈11 kPa, with the higher values associated with finer particles. However, if sand contains a significant amount of silt or clay, a higher *AEV* can be obtained. Therefore, the simulated soils in this study may represent sand with a smaller *AEV* or sand with a small portion of fines (silt and clay), resulting in a higher *AEV*.

## 3. Problem geometry and materials

**Fig 4:**
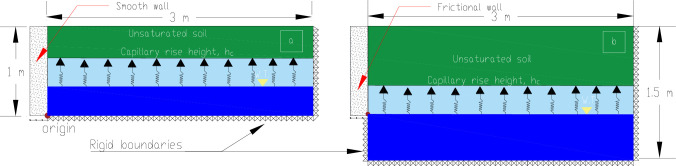
Schematic diagrams for the (a) smooth wall and (b)frictional wall.

The schematic diagrams for modeled rigid concrete walls are shown in Fig 4. To address potential failure mechanism restrictions, it was necessary to extend the boundary 0.5 m below the base of the frictional walls. The numerical results were normalized over the height of the wall (H = 1 m). The arbitrarily selection of the wall’s height, therefore, was not an issue. Table 1 displays the parameters required in the simulation using the UNSAT-DLO approach along with the suggested wall friction values (δ). The capillary rise height, h_c_, was considered above the water table level (H_w_), see Fig 4, calculated using h_c_ = *AEV*/*γ*_*w*_, *γ*_*w*_ is the unit weight of water. Above the capillary rise height, the backfill material was assumed to be unsaturated where the suction head defined as the distance from H_w_ to a specific level above h_c_. In the simulation, suction was accomplished by lowering the position of H_w_.

**Table 1:** Required parameters in the UNSAT-DLO approach and for the Fredlund and Rahardjo (1993) equation.

*γ*_*sat*_		*γ*_*dry*_		*γ*_*unsat*_		*c*		*ϕ*		*κ*		*AEV*		h_c_		Wall friction	
(kN/m^3^)		(kN/m^3^)		(kN/m^3^)		(kPa)		(degrees)		(kPa^-1^)		(kPa)		(m)		δ (degrees)	
20		15		17.5		0		30		0.05		5		0.509		0,0.33,0.5,0.67,1	
20		15		17.5		0		30		0.05		10		1.019		0,0.33,0.5,0.67,1	
20		15		17.5		0		30		0.05		15		1.529		0,0.33,0.5,0.67,1	
20		15		17.5		0		30		0.05		20		2.038	0,0.33,0.5,0.67,1		
20		15		17.5		0		30		0.05		30		3.058	0,0.33,0.5,0.67,1		

## 4. Numerical results

### 4.1 The effect of air entry value on passive earth thrust

**Fig 5:**
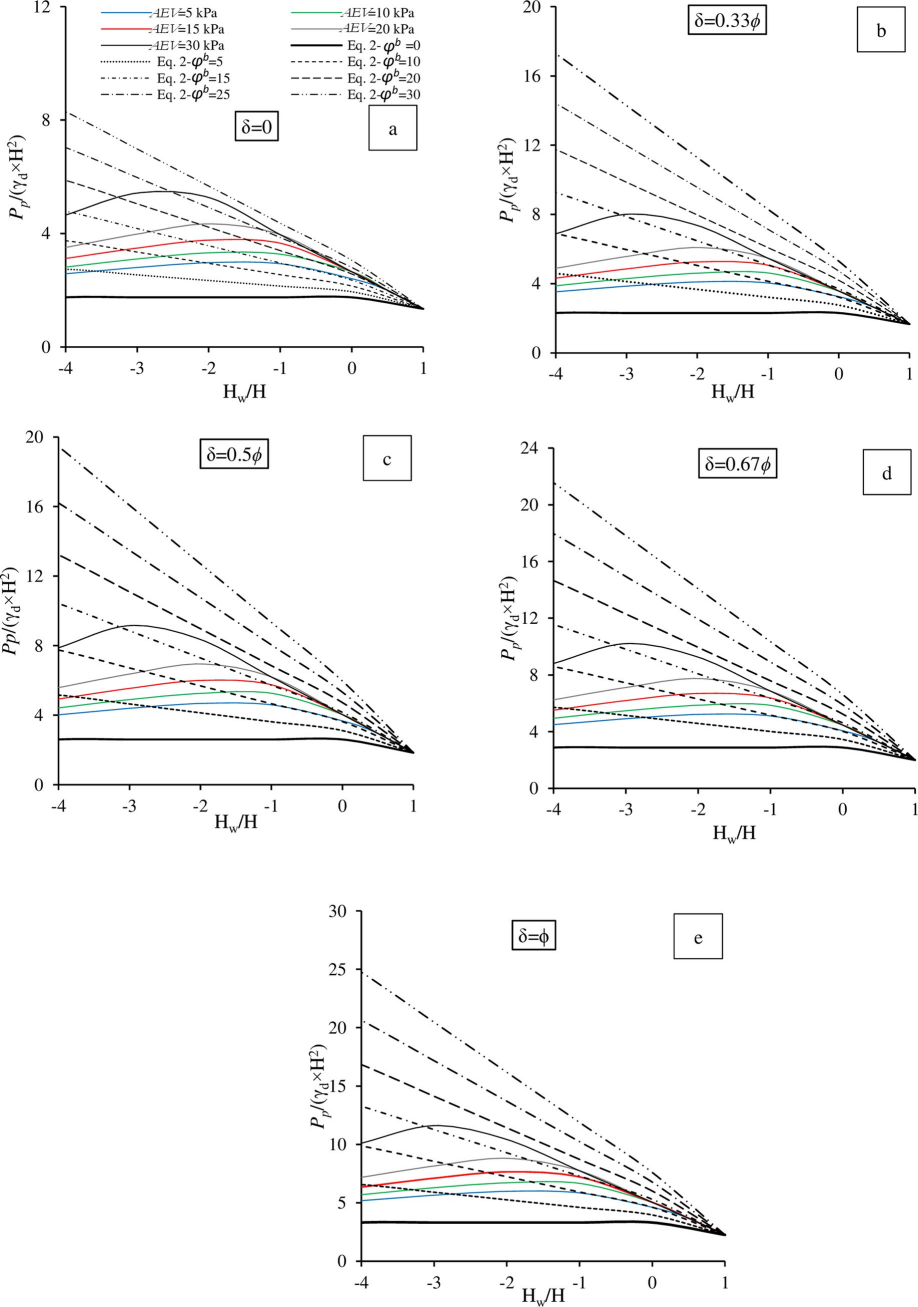
Design charts for the obtained *P*_*p*_ for various *AEV* at various wall frictions for (a) δ = 0, (b) δ = 0.33 *ϕ*, (c) δ = 0.5 *ϕ*, (d) δ = 0.67 *ϕ* and (e) δ = *ϕ*.

The design charts obtained for this series are shown in Fig 5. Normalized results for a range of *AEV* and δ values were compared with the Fredlund and Rahradjo (1993) equation for a smooth wall (δ = 0). The equation accounts the effect of suction, originally extended based on the Rankine approach, as stated previously.

$$P_{p}=[\frac{1}{2}k_{p}\gamma^{\prime }H^{2}+2c^{\prime }\sqrt{k_{p}}H+2(u_{a}-u_{w})\tan\phi^{b}\sqrt{k_{p}}H]$$ (2)

where *P*_*p*_ is the exerted effective passive thrust, *k*_*p*_ is the passive lateral earth pressure coefficient, *γ*′ is the effective unit weight, *H* is the height of the wall, *c*′ is the effective cohesion, *u*_*a*_ is the pore air pressure, *u*_*w*_ is the pore water pressure and *ϕ*^*b*^ is the angle of friction with respect to changes in matric suction. Eq 2 is similar to an equation available for passive earth pressure problems (accounting for unsaturated conditions) derived by Lu and Likos (2004) based on the Rankine approach. The equation by Lu and Likos (2004) is based on Bishop’s parameter, χ, which accounts for different soil types.

In the analysis, various values of *ϕ*^*b*^ were used (0, 5, 10, 15, 20, 25 and 30°). Rassam and Cook [49] also suggested various magnitudes of *ϕ*^*b*^ according to *AEV*. Apart from Eq 1, the numerical analysis also took into consideration the influence of capillary rise, as mentioned previously. This exposed the classical assumption of dismissing the effect of the capillary rise above the water table. *k*_*p*_ values were obtained according to δ, shown in Table 1. Antão et al. [50] proposed a series of *k*_*p*_ on the basis of δ, which were used in Eq 2. The values of *k*_*p*_ at *ϕ* = 30° were 3.00, 3.9632, 4.4610, 4.9435 and 5.6786 for δ values of 0, 0.33 *ϕ*, 0.5 *ϕ*, 0.67*ϕ* and δ = *ϕ*, respectively.

The simulation results, as depicted in Fig 5, revealed non-linear trends, primarily influenced by saturation conditions. In contrast, Eq 2 demonstrated an almost linear trend. This discrepancy was attributed to the presumed linear relationship between suction and *P*_*p*_ in Eq 2. Generally, the numerical results demonstrated a significant influence of unsaturated conditions. An increase of 1.79-fold in *P*_*p*_ was obtained when H_w_ dropped from 1 m (fully saturated case) to 0 m (at the base of the wall) for δ = 0 at *AEV* = 5 kPa. The increase became even more pronounced with a further drop in H_w_, e.g., 2.21-fold at H_w_ = -2 m.

It was intriguing to observe that the effect of the *AEV* using the UNSAT-DLO approach was significant. The higher *AEV*, the higher *P*_*p*_. The increase in *P*_*p*_ for δ = 0 at *AEV* = 30 kPa was by 4-fold when H_w_ dropped 3 m from the surface compared with the fully saturated condition. This was higher by 1.94-fold of its counterpart for *AEV* = 5 kPa. This indicated the considerable effect of *AEV*. This increase was even higher for the frictional walls, e.g., reaching a 2-fold difference for δ = *ϕ*. As stated previously, the change of *AEV* was considered in this study to represent hydraulic (drying and wetting cycles) hysteresis and/or mechanical hysteresis of the SWRC. Consequently, the effect of hydraulic or mechanical hysteresis on *P*_*p*_ was found to be significant.

The maximum *P*_*p*_ was obtained just beyond the *AEV* values, with a further shift towards higher suction noted for the higher *AEV*. This marked the transition zone between the *AEV* and the residual suction. The was mainly attributed to the effective transmission of suction by water content to the soil particles. Beyond this threshold, a significant reduction in *P*_*p*_ was obtained, attributed to the strength reduction induced desaturation for the simulated sands within the modeled suction profiles. In other words, due to the significant reduction in water content, suction was not effectively transferred to the reduced aggregate particles. Cohesion enhancement or suction stress-induced shear strength in clays has been shown to increase monotonically. However, the results obtained demonstrated a non-monotonic relationship (reduction after peak), where suction reached high levels while the effective contact surface was low due to minimal soil moisture. This often occurs in sand or sand-fine mixtures with a major proportion of sand particles. A reduction of 13.18% in *P*_*p*_ was obtained due to desaturation when H_w_ dropped from -2 to -4 kPa for δ = 0 and *AEV* = 5 kPa. It can be noted that Eq 2 did not take into consideration the effect of desaturation, as the trends were linear. In addition, although the assumed values of *ϕ*^*b*^ were reasonably defined among the obtained results of the UNSAT-DLO approach, the results of Eq 2 were overestimated for the frictional walls.

Table 2 summarizes, based on the results from Fig 5, the rate of increase in *P*_*p*_ at different H_w_ in comparison to the fully saturated case (H_w_ = 1m) for δ = 0. As stated earlier, the influence of unsaturation conditions on *P*_*p*_ was found to be significant. Additionally, the maximum *P*_*p*_ was observed at H_w_/H = -2 (see Table 2), except for the case with *AEV* = 30 kPa. The significance of the results presented in Table 2 lies in their relevance to the impact of suction on the stability of numerous structures. These structures often gain additional strength from unsaturated conditions. Considering that soils frequently exist in unsaturated states, neglecting the influence of suction has led to an underestimation of the stability of various geotechnical structures, (2004).

**Table 2:** Rate of increase in *P*_*p*_ for smooth wall, δ = 0 compared with the fully saturated case, H_w_ = 1m.

	*AEV *(kPa)				
H_w_/H	5	10	15	20	30
0.5	1.49	1.49	1.49	1.49	1.49
0	1.79	1.97	1.97	1.97	1.97
-1	2.18	2.43	2.72	2.94	2.94
-2	2.21	2.47	2.8	3.22	3.92
-3	2.08	2.31	2.59	2.96	4.03
-4	1.92	2.09	2.32	2.61	3.46

### 4.2 The effect of wall friction on passive earth thrust

**Fig 6:**
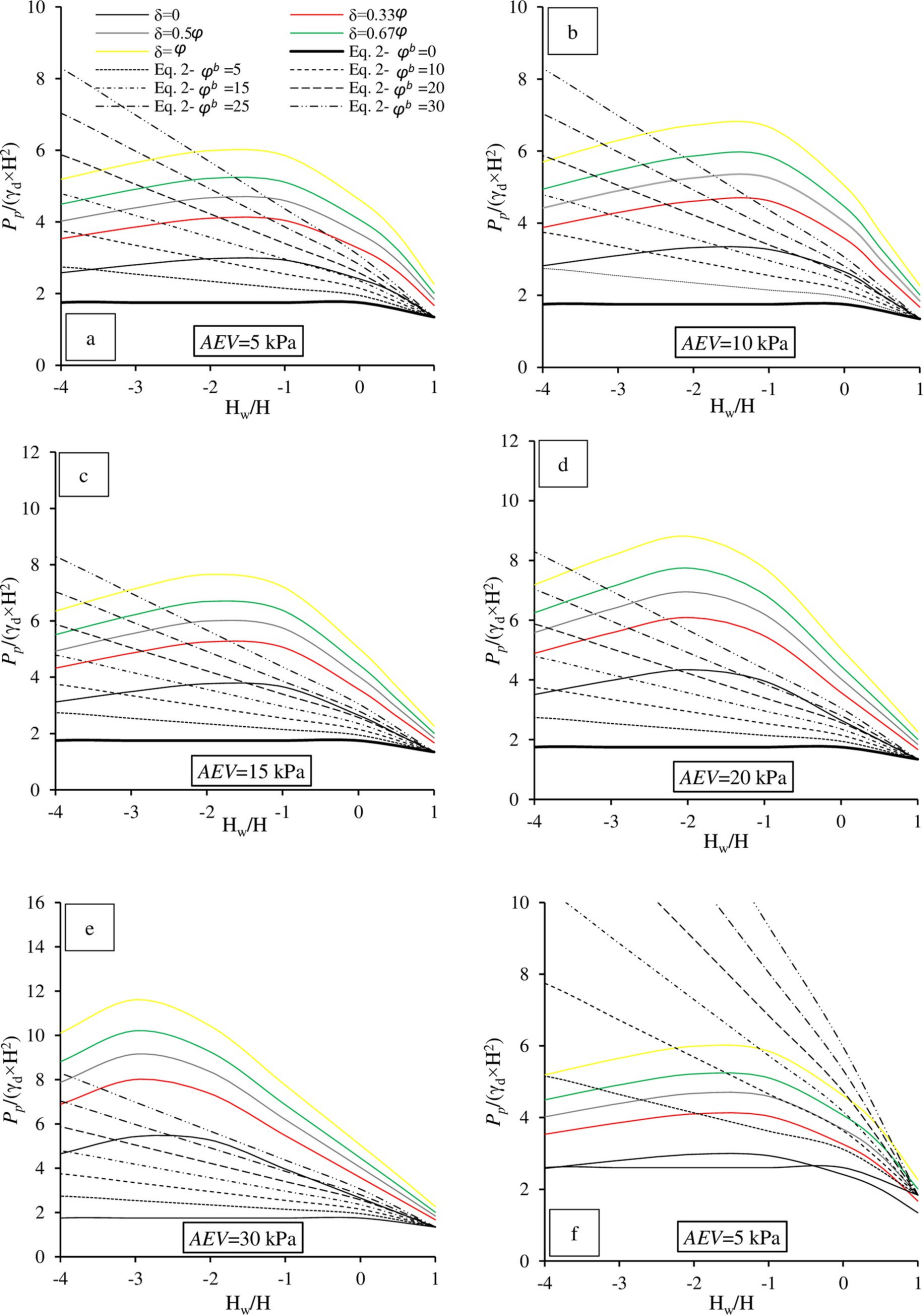
Design charts for the obtained *P*_*p*_ for various δ (Eq 2 fitted at δ = 0) for *AEV =* (a) 5 kPa, (b) 10 kPa, (c) 15 kPa, (d) 20 kPa, (e) 30 kPa and (f) *AEV =* 5 kPa for Eq 2 fitted at δ = 0.5 *ϕ*.

The design charts of this series exhibited non-linear trends as depicted in Fig 6. The numerical results were also compared with those obtained from Eq 2 at different δ values. As it was expected, the higher frictional of the wall, the higher *P*_*p*_ obtained. For example, at *AEV* = 10 kPa and H_w_ = -1 m, an increase in *P*_*p*_ of 2-fold was obtained when δ = *ϕ* was compared with δ = 0. As stated previously, the influence of unsaturated conditions on *P*_*p*_ can be readily observed. For example, at *AEV* = 15 kPa and δ = 0.67 *ϕ*, an increase of about 3.32-fold in *P*_*p*_ was obtained when H_w_ dropped from 1 m to -2 m. This increase was approximately 1.19 times higher than the increase observed for δ = 0 at the same H_w_ drop. This implied the effect of suction stress on *P*_*p*_ at different δ values. On the other hand, Eq 2 underestimated the numerical results because its results were fitted at δ = 0. However, it overestimated the numerical results when cases with δ ≥ 0.5 *ϕ* were considered, as shown in Fig 6F.

Table 3 provides insights into the rate of increase in *P*_*p*_ for different δ compared with the δ = 0 at three H_w_ values, with a focus on the maximum rates achieved. The data underscores the considerable effect of δ when H_w_ remains constant. For example, at H_w_/H = -2 and *AEV* = 5 kPa, an increase of about 2.01-fold (see Table 3) in *P*_*p*_ was obtained when δ = *ϕ* was compared with δ = 0. The implication is important as it highlights the significance of understanding and accounting for δ in the analysis of passive thrust under unsaturated conditions. Wall friction plays a crucial role in determining the stability and behavior of structures [51]. By examining the rates of increase under various wall frictions, designers can better assess structures.

**Table 3:** Rate of increase in *P*_*p*_ for different wall roughness compared with the smooth wall (δ = 0).

		*AEV*				
Wall roughness	H_w_/H	5	10	15	20	30
δ = 0.33 *ϕ*	-2	1.38	1.39	1.4	1.4	1.4
	-3	1.38	1.38	1.39	1.4	1.48
	-4	1.37	1.38	1.38	1.39	1.48
δ = 0.5 *ϕ*	-2	1.57	1.58	1.59	1.6	1.59
	-3	1.57	1.58	1.59	1.6	1.69
	-4	1.56	1.57	1.58	1.59	1.69
δ = 0.67 *ϕ*	-2	1.75	1.77	1.78	1.78	1.76
	-3	1.75	1.76	1.78	1.79	1.88
	-4	1.74	1.75	1.77	1.78	1.89
δ = *ϕ*	-2	2.01	2.02	2.03	2.03	1.98
	-3	2.02	2.03	2.04	2.05	2.14
	-4	2.01	2.02	2.03	2.05	2.17

### 4.3 Effect of air entry value versus wall friction on passive earth thrust

**Fig 7:**
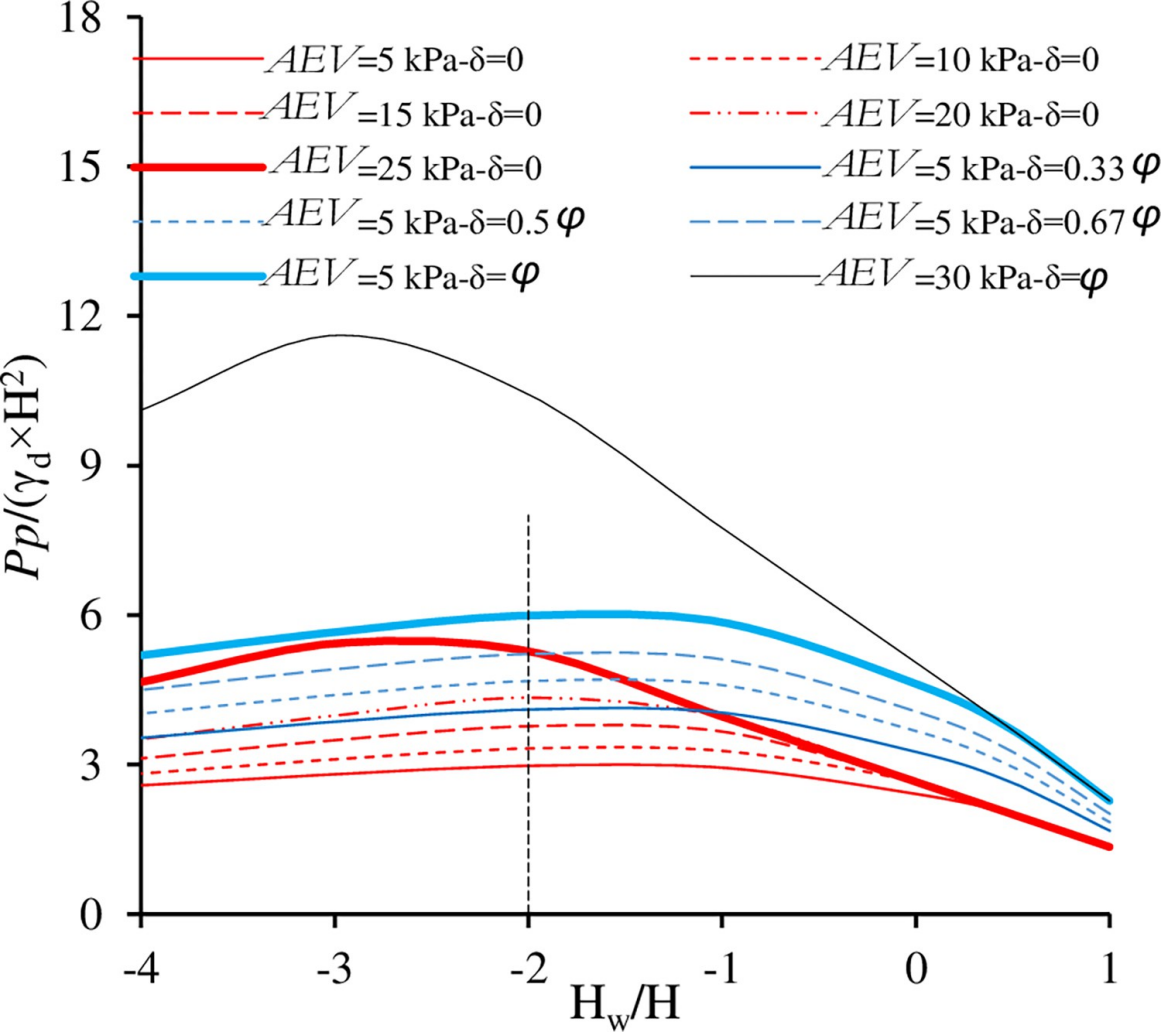
The effect of *AEV* versus the effect of the wall roughness (δ) on *P*_*p*_.

Fig 7 shows a comparison between the effects of *AEV* (red curves) due to hydraulic and mechanical hysteresis at a constant δ value and the effects of wall friction δ (blue curves) at a constant *AEV* of 5 kPa.

As stated previously, the effect of both *AEV* and δ on *P*_*p*_ was significant. The effect of δ was more pronounced at low suction until about H_w_/H = -2 m (equivalent to *s* = 29.4 kPa). Further drop in H_w_ (> -2 m) caused the effect of *AEV* to override the influence of wall roughness for all δ values, except for the fully frictional wall (δ = *ϕ*). The implication of this effect is highly significant, as any change in *AEV* resulting from hydraulic (scenarios involving cycles of drying and wetting) and mechanical hysteresis can impact wall stability, irrespective of wall roughness. From a practical standpoint, it is essential to incorporate an understanding of the hydraulic and mechanical hysteresis phenomenon into the analysis and design process[52]. This assessment seeks to ascertain whether current geotechnical infrastructures might be conservative, prompting the necessity for an adjusted understanding in light of these phenomena’s effects.

Fig 7 also show the mutual effect of both *AEV* and δ on *P*_*p*_ (the black curve) where their effects were seen to be very significant. An increase of 4.14-fold in *P*_*p*_ at H_w_/H = -3 was obtained when comparing the case *AEV* = 30 kPa and δ = *ϕ* with *AEV* = 5 kPa and δ = 0.

## 5. Conclusions

Hydraulic and mechanical hysteresis are key features of the soil water retention curve (SWRC). Although SWRC hysteresis has been extensively studied in previous research (e.g., [26–28, 31, 32], a significant gap remains in the literature regarding the application of SWRC hysteresis in the analysis of passive earth pressure.

This paper reports numerical studies on the effects of hydraulic (drying and wetting cycles) and mechanical hysteresis on passive earth pressure. Parametric studies were conducted using the upper bound theorem at various air entry values (*AEV*) ranging from 5 kPa to 30 kPa and for different frictional walls: δ = 0, 0.33 *ϕ*, 0.5 *ϕ*, 0.67 *ϕ* and δ = *ϕ*. The modeled suction ranged from zero to the transition zone, with the maximum applied suction significantly influenced by the *AEV*.

The numerical results were compared with those of an analytical method based on the limit equilibrium available in the literature. The following conclusions were drawn:

1. A set of design charts for passive earth pressure problems was proposed, taking into account the hydraulic and mechanical hysteresis of the SWRC across various soil strengths, wall frictions, and suction profiles for a rigid concrete retaining wall.
2. A non-linear relationship between the effective passive thrust (*P*_*p*_) and suction was obtained where the effect of the hydraulic and mechanical hysteresis on *P*_*p*_ was significant. An increase of 1.77-fold in *P*_*p*_ was obtained for the frictional wall (δ = 0) when *AEV* increased from 5 kPa to 30 kPa for the water table (H_w_ = -2m), 2 m below the base of the wall.
3. The expected influence of δ on *P*_*p*_ was also significant. However, it was observed that hydraulic and mechanical hysteresis impart an additional strength to *P*_*p*_, well in excess of what can be obtained solely through wall friction. For example, the 1.77-fold increase in *P*_*p*_ obtained for δ = 0, when *AEV* increased from 5 kPa to 30 kPa at H_w_ = -2 m, was higher by 1.12-fold than the increase observed when the wall friction was changed from δ = 0 to δ = 0.5 *ϕ* at *AEV* = 5 kPa.
4. The necessity for the proposed design charts for retaining structures, considering hydraulic and mechanical hysteresis, becomes essential. The implications of such design charts are significant, as they have the potential to assist in design considerations in scenarios involving partial saturation, soil strength, and wall friction.

Although the numerical results were validated against the analytical approach, an existing passive earth pressure equation, this research emphasizes a limitation, indicating the necessity for additional validations using experimental data that consider hydraulic and mechanical hysteresis. Consequently, it would be advisable to await further validation of the method until experimental or field data become available. Additionally, this study did not account for the transient seepage of soil water through the backfill material behind the wall.
